# Influence of Rainfall on *Leptospira* Infection and Disease in a Tropical Urban Setting, Brazil

**DOI:** 10.3201/eid2602.190102

**Published:** 2020-02

**Authors:** Kathryn P. Hacker, Gielson A. Sacramento, Jaqueline S. Cruz, Daiana de Oliveira, Nivison Nery, Janet C. Lindow, Mayara Carvalho, Jose Hagan, Peter J. Diggle, Mike Begon, Mitermayer G. Reis, Elsio A. Wunder, Albert I. Ko, Federico Costa

**Affiliations:** University of Pennsylvania, Philadelphia, Pennsylvania, USA (K.P. Hacker);; Yale University, New Haven, Connecticut, USA (K.P. Hacker, J.C. Lindow, J. Hagan, E.A. Wunder, Jr., A.I. Ko, F. Costa);; Fundação Oswaldo Cruz, Salvador, Brazil (G.A. Sacramento, J.S. Cruz, D. de Oliveira, N. Nery, Jr., J.C. Lindow, M. Carvalho, J. Hagan, M.G Reis, A.I. Ko, F. Costa);; Montana State University Bozeman, Bozeman, Montana, USA (J.C. Lindow); Lancaster University, Lancaster, UK (P.J. Diggle);; Johns Hopkins University, Baltimore, Maryland, USA (P.J. Diggle);; University of Liverpool, Liverpool, UK (M. Begon);; Universidade Federal da Bahia, Salvador (M.G. Reis, F. Costa)

**Keywords:** leptospirosis, *Leptospira*, bacteria, public health, epidemiology, urban epidemiology, rainfall, waterborne infections, seasonal infection, temporal dynamics, slum health, zoonoses, Brazil

## Abstract

The incidence of hospitalized leptospirosis patients was positively associated with increased precipitation in Salvador, Brazil. However, *Leptospira* infection risk among a cohort of city residents was inversely associated with rainfall. These findings indicate that, although heavy rainfall may increase severe illness, *Leptospira* exposures can occur year-round.

Leptospirosis, a leading zoonotic cause of illness and death (*1*), has emerged as a major health problem due to the global expansion of urban slum communities (*2**–**4*). The disease is associated with severe manifestations such as Weil’s disease and pulmonary hemorrhage syndrome (*5*), for which case-fatality rates are 10%–50% or even higher (*6*). Transmission to slum residents occurs in the peridomiciliary environment, in which exposures to sewers, floodwater, and contaminated soil are risk factors (*3**,**7**,**8*). Extreme weather events may precipitate outbreaks (*3**–**6*), as recently experienced during the aftermath of Hurricane Maria in Puerto Rico (*9*). Similarly, seasonal periods of heavy rainfall and flooding are a contributing factor to the risk for urban leptospirosis (*4**,**10*).

In urban slum settings, contact with rats and *Leptospira*-contaminated water and soil occur year-round (*3*). Prior studies have shown, consistently, positive associations between heavy rainfall and hospitalized leptospirosis case-patients (*4**,**10*). However, this relationship may be affected by differences in case definitions used by diverse surveillance systems. In the few prospective cohort studies available, estimates of severe disease accounted for only a small proportion of the total disease burden (*6*). Thus, little is known about the role of rainfall in overall infection rates. To characterize the seasonal pattern of leptospirosis and *Leptospira* infection in a tropical urban setting and evaluate the influence of meteorological factors on seasonal risk, we conducted a prospective investigation of *Leptospira* infection rates among slum residents while actively surveying for hospitalized leptospirosis case-patients within Salvador, Brazil, during seasonal periods of high and low rainfall.

## The Study

During February 2013–April 2015, we identified patients >5 years old with suspected leptospirosis at the state infectious disease hospital in Salvador, Brazil (*4**,**5*), and those reported in the public health surveillance database by other hospitals in Salvador. We estimated the probable date of infection as 15 days before the hospital admission date. We evaluated suspected leptospirosis cases according to the WHO case definition standard (*4**,**6**,**11*) using the microscopic agglutination test (MAT), *lipL32* real-time PCR assay (*11*), IgM-ELISA (*6*), or a combination. We defined laboratory-confirmed cases of leptospirosis as those with >4-fold rise in MAT titers in paired serum samples, MAT titers >1:800 in a single sample, or positive PCR (Appendix Tables 1, 2).

**Figure 1:**
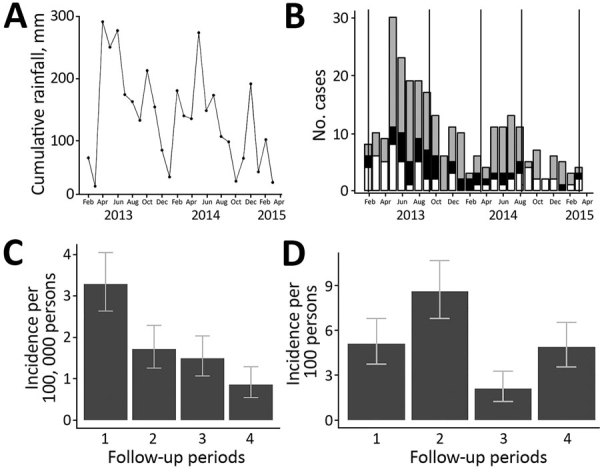
Temporal distributions of rainfall, cases of leptospirosis requiring hospitalization, and *Leptospira* infections in Salvador, Brazil, February 2013–March 2015. A) Cumulative monthly rainfall. B) Monthly citywide cases of leptospirosis requiring hospitalization, which were reported to the national surveillance system and stratified according to confirmed (black bar), probable (gray bars), and unconfirmed (white bars) case status. Vertical lines represent the dates the 5 serosurveys were performed during the 2-year study. C) Cumulative incidence of citywide cases of leptospirosis requiring hospitalization during 4 biannual follow-up periods for a community-based cohort. D) Cumulative incidence of *Leptospira* infection among a cohort of 861 residents of an urban slum community within Salvador during 4 biannual follow-up periods. Error bars in panels C and D indicate 95% CIs.

**Figure 2:**
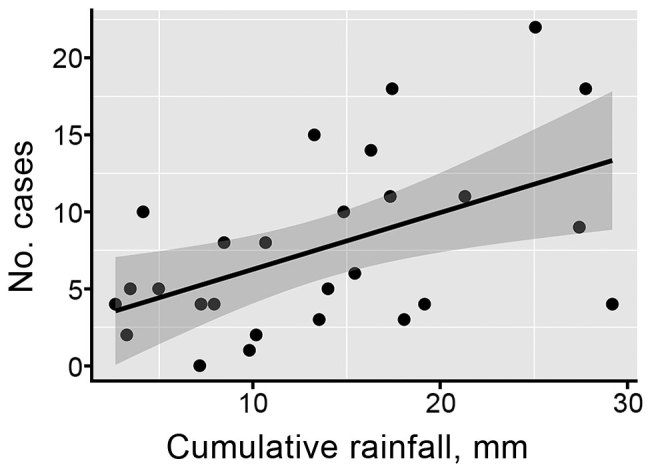
Correlation between cumulative monthly rainfall and monthly citywide cases of leptospirosis requiring hospitalization.

A linear regression model identified that cumulative monthly rainfall (Figure 1, panel A) was significantly associated with the monthly number of hospitalized cases (r^2^ = 0.22, p<0.007) (Figure 2). The highest hospitalized disease incidence occurred during the first period (February–September 2013; 3.29 cases/100,000 population; 95% CI 2.67–4.01 cases/100,000 population) and decreased across the next periods (Table 1; Figure 1, panels B, C).

**Table 1:** Cumulative rainfall, citywide incidence of leptospirosis requiring hospitalization, and incidence of *Leptospira* infection among a community-based cohort in Salvador, Brazil, 2013–2015*

Follow-up period (dates)*	Cumulative rainfall, cm (+ SD)†	Hospitalizations/100,000 population‡			*Leptospira* infection in period§	
		No. cases	Incidence (95% CI)		No. infected	Incidence (95% CI)
1 (2013 Feb 2–Sep 10)	126 (+ 13)	88	3.29 (2.67–4.01)		44	5.11 (3.74–6.80)
2 (2013 Sep 10–2014 Mar 14)	81 (+ 21)	46	1.72 (1.26–2.29)		74	8.60 (6.81–10.67)
3 (2014 Mar 14–2014 Aug 8)	93 (+ 16)	40	1.50 (1.07–2.04)		18	2.09 (1.24–3.28)
4 (2014 Aug 8–2015 Mar 3)	57 (+ 11)	23	0.86 (0.54–1.29)		42	4.88 (3.54–6.54)

*We conducted 5 semiannual follow-up surveys for a community-based cohort of 861 residents of a community within Salvador, Brazil. A period was defined as the interval between 2 consecutive surveys. †The source of rainfall data is 4 weather stations maintained by the Brazilian Institute for the Environment and Water Resources (Instituto do Meio Ambiente e Recursos Hidrilcos), located 1.6 km from the study site. ‡Cases of hospitalized leptospirosis per 100,000 population in the city of Salvador, Brazil (pop. 2,675,656 in 2010), during the follow-up period. §We performed microscopic agglutination test to evaluate serologic evidence of *Leptospira* infections between 2 consecutive surveys. Cumulative incidence was calculated as the number of infections per 861 cohort subjects multiplied by 100.

Concurrently, we conducted a prospective cohort study assessing serologic evidence of *Leptospira* infection among urban slum residents of Pau da Lima, northwestern Salvador. We enrolled 2,421 of 3,716 eligible residents, >5 years of age and with written informed consent, of whom 821 participated in all serologic surveys performed twice annually during August–September (dry season) and February–March (rainy season) during 2013–2015 (Figure 1, panel A). Using panels with the 2 most common *Leptospira* species in Salvador (*4*), *L. interrogans* serogroup Icterohaemorrhagiae serovar Copenhageni (strain Fiocruz L130) and *L. kirsheri* serogroup Cynopteri serovar Cynopteri (strain 3522C), we defined serologic evidence of *Leptospira* infection by a MAT titer increase from negative to >1:50 (seroconversion) or >4-fold increase between sequential, paired samples. During the study period, 29% of the infected participants reported fever.

To assess the association between rainfall and laboratory-confirmed *Leptospira* infection, we calculated the cumulative amount of rainfall that each study participant experienced between sequential samples. We used a generalized estimating equation and incorporated explanatory variables for gender, age, time period, and cumulative rainfall that each participant experienced. In contrast to the hospitalized cases, we found *Leptospira* infection risk in the urban area had an inverse association with cumulative rainfall (0.986 cm, 95% CI 0.977–0.995 per cm) (Table 2; Figure 1, panel D). We additionally assessed various rainfall metrics, as well as the number of severe rainfall events each participant experienced above the mean rainfall, and the resulting patterns remained consistent. Increasing age and male sex were associated with higher infection risk.

**Table 2:** Association of cumulative rainfall and semiannual follow-up period with risk for *Leptospira* infection, Salvador, Brazil, 2013–2015*

Variable	Odds ratio (95% CI)
Per year of age	1.02 (1.02–1.03)
Male sex	1.98 (1.48–2.64)
Cumulative rainfall, cm†	0.986 (0.977–0.995)
Period	
1	Referent
2	1.15 (0.63–2.10)
3	0.30 (0.15–0.59)
4	0.44 (0.20–0.97)

*We used Generalized Estimating Equation to evaluate the association of rainfall, follow-up period, and patient age and sex on *Leptospira* infection, as ascertained by serologic evidence, assuming a dependence on the individual level across the 4 repeated measures. †Cumulative amount of rainfall experienced by participant between sequential samples.

## Conclusions

Leptospirosis is traditionally associated with heavy rainfall and flooding events in Brazil (*5**,**9*) and worldwide (*7**,**10*). Our findings support the association between extreme weather events and clinical leptospirosis. During the study period, the risk of acquiring leptospirosis that required hospitalization was significantly higher in periods with elevated rainfall. However, this finding is in contrast to *Leptospira* infection in nonhospitalized persons.

Our findings indicate that *Leptospira* infections occur year-round in this urban tropical setting and the cumulative incidence of *Leptospira* infection is high (2%–9% per period). This finding differs from patterns that we and others have identified for leptospirosis requiring hospitalization (*2**,**4**,**9**,**12*). Although this study does not specifically assess subclinical symptomatic infection, it provides further evidence that the impact of leptospirosis is underestimated, and physicians should be aware that leptospirosis infection may manifest clinically year-round.

The patterns of *Leptospira* exposure incidence and infection severe enough to require hospitalization, when taken together, suggest that rainfall may promote exposures of greater inocula, which in turn may increase the risk of developing severe clinical outcomes, such as severe pulmonary hemorrhage syndrome and Weil’s disease. For example, heavy rainfall may diffuse *Leptospira* from the soil, resulting in higher concentrations of bacteria in the media to which humans are exposed (sewer water) and so to a higher inoculum dose, thus increasing hospitalized disease incidence and perhaps decreasing the environmental exposure risk in and around households (mud and exposed soil) and decreasing infection risk. However, additional studies are needed to assess the specific contribution of inoculum dose to disease severity.

The 2-year study period was atypical because rainfall was lower than expected during the rainy seasons (Figure 1, panel A; Appendix Figure 1). Of note, we observed a significant inverse association between cumulative rainfall and the risk for infection during biannual sampling periods. Thus, these trends may not apply to periods with higher amounts of rainfall or extreme climatic events, such as El Niño. This study was also limited because we used seroconversion to identify infection and therefore could not determine the precise timing of exposure events; furthermore, we conducted serologic surveys only in a single urban slum community. However, most hospitalized cases occur in similar communities (*4*), and therefore Pau da Lima is likely to be representative. Last, although the surveillance hospitals were able to capture a variety of febrile illnesses, they did not capture mild febrile illness, which may account for a missing proportion of leptospirosis cases.

Our findings demonstrate that, despite the association of leptospirosis hospitalization with rainfall, *Leptospira* exposure continues year-round. Although we did not evaluate mild subclinical or clinical infections, it is possible that participants experience symptomatic illness that may be unrecognized or misdiagnosed as dengue or other febrile disease (*12**,**13*). Clinicians should be aware that leptospirosis may manifest clinically outside of normal seasonal periods of heavy rainfall. In addition, the differences observed during the time periods independent from rainfall indicate that other unexplained factors may influence the temporal risk for *Leptospira* infection. Identifying these factors will help enhance intervention strategies in urban slum environments.

Appendix Additional information about the influence of rainfall on *Leptospira* infection and disease in a tropical urban setting, Brazil.
