# Supplementary material for: Influence of Rainfall on Leptospira Infection and Disease in a Tropical Urban Setting, Brazil
Source: Emerg Infect Dis. 2020 Feb;26(2):311–4. doi: 10.3201/eid2602.190102 (PMC6986844; doi:10.3201/eid2602.190102)
Supplement: Appendix — Additional information about the influence of rainfall on Leptospira infection and disease in a tropical urban setting, Brazil. [file 19-0102-Techapp-s1.pdf]

Article DOI: https://doi.org/10.3201/eid2602.190102

Influence of Rainfall on *Leptospira* Infection and Disease in a Tropical Urban Setting, Brazil

Appendix

Appendix Table 1. Diagnostic criteria of confirmed-positive cases of leptospirosis requiring hospitalization from 2 reference hospitals in Salvador, Brazil, January 2013–April 2015*

No. cases confirmed positive (%) N = 161†	Culture	MAT‡	ELISA 1§	PCR
60 (37.3)	Negative	Positive	Positive	Negative
23 (14.2)	Negative	Positive	NA	Negative
18 (11.2)	Negative	Positive	Positive	NA
17 (10.6)	Negative	Positive	Positive	Positive
7 (4.3)	Negative	Negative	NA	Positive
7 (4.3)	Negative	Negative	Negative	Positive
4 (2.5)	Negative	Positive	NA	Positive
4 (2.5)	Negative	Positive	Negative	Negative
2 (1.2)	Positive	Positive	NA	Positive
2 (1.2)	Positive	Negative	NA	Positive
2 (1.2)	Positive	Negative	NA	Negative
2 (1.2)	NA	Positive	NA	Positive
2 (1.2)	NA	Positive	Positive	Negative
2 (1.2)	Positive	Positive	Positive	Positive
2 (1.2)	Negative	Positive	Negative	Positive
2 (1.2)	NA	Positive	Positive	NA
1 (0.62)	Positive	Positive	NA	Negative
1 (0.62)	NA	Positive	NA	Negative
1 (0.62)	Positive	Positive	Negative	Positive
1 (0.62)	Negative	Negative	Positive	Positive
1 (0.62)	Positive	Positive	Positive	NA

*Positive samples were defined as a ≥4-fold rise in MAT titers of paired serum samples, MAT titers ≥1:800 in a single sample, or positive PCR. Diagnostic tests are marked as NA if the diagnostic test was not performed. MAT, microscopic agglutination test; NA, not available.

†Total number of confirmed positive symptomatic hospitalized patients from the 2 reference hospitals based on panel of diagnostic tests.

‡MAT positivity was determined as having titers ≥1:800 in a single sample or a ≥4-fold rise in MAT titers for paired serum samples.

Appendix Table 2. Characteristics of confirmed positive hospitalized cases from 2 reference hospitals in Salvador, Brazil, January 2013–April 2015

Characteristic	No. responses	Mean (SD) or % of group
Clinical symptoms		
No. days of symptoms before admission to hospital	161	7.2 (3.5)
Fever	161	100.0%
Conjunctival suffusion	159	8.8%
Jaundice	160	73.8%
Complications during hospital stay		
Oliguria	160	34.4%
Hemoptysis	159	34.0%
Respiratory insufficiency	161	24.8%
Arrhythmia	141	11.3%
Altered mental status	161	5.0%
Outcome		
Days spent in hospital	161	11.2 (10.4)
Case-fatality rate	161	7.5%

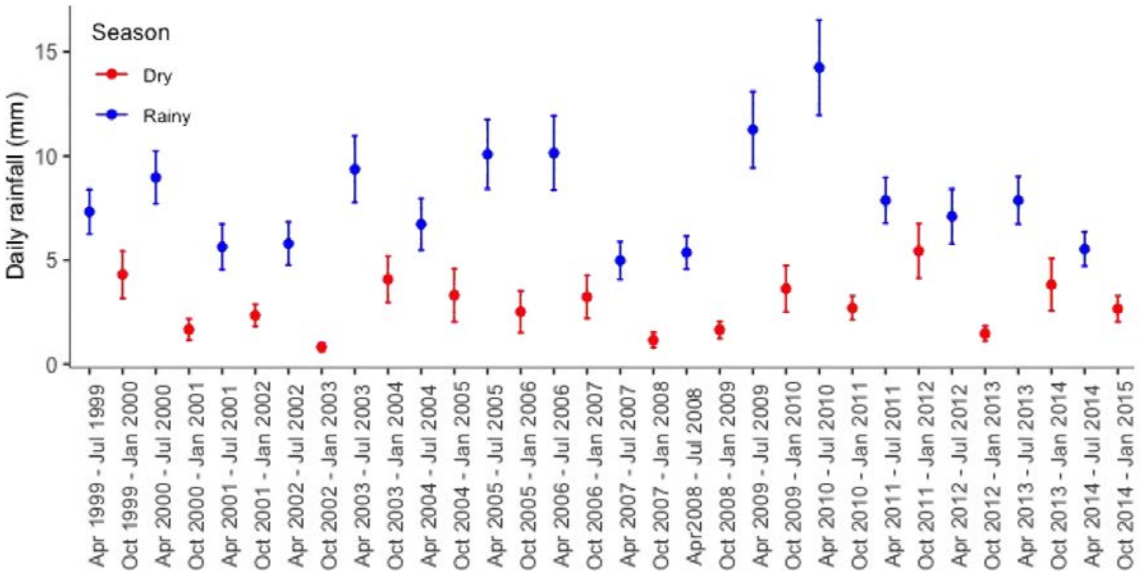

Appendix Figure. Seasonal cumulative rainfall in Salvador, Brazil, 1999–2015. The rainy season typically occurs April–July (red) and the dry season October–January (red). Black vertical lines denote the start of the seasonal follow-up periods evaluated in the study.
